# Alternative splicing during fruit development among fleshy fruits

**DOI:** 10.1186/s12864-021-08111-1

**Published:** 2021-10-26

**Authors:** Xiaomin Yan, Dan Bai, Hongtao Song, Kui Lin, Erli Pang

**Affiliations:** MOE Key Laboratory for Biodiversity Science and Ecological Engineering and Beijing Key Laboratory of Gene Resource and Molecular Development, College of Life Sciences, Beijing Normal University, No 19 Xinjiekouwai Street, Beijing, 100875 China

**Keywords:** Fleshy fruits, Alternative splicing, Immature stage, Ripe stage

## Abstract

**Background:**

Alternative splicing (AS) is an important mechanism of posttranscriptional modification and dynamically regulates multiple physiological processes in plants, including fruit ripening. However, little is known about alternative splicing during fruit development in fleshy fruits.

**Results:**

We studied the alternative splicing at the immature and ripe stages during fruit development in cucumber, melon, papaya and peach. We found that 14.96–17.48% of multiexon genes exhibited alternative splicing. Intron retention was not always the most frequent event, indicating that the alternative splicing pattern during different developmental process differs. Alternative splicing was significantly more prevalent at the ripe stage than at the immature stage in cucumber and melon, while the opposite trend was shown in papaya and peach, implying that developmental stages adopt different alternative splicing strategies for their specific functions. Some genes involved in fruit ripening underwent stage-specific alternative splicing, indicating that alternative splicing regulates fruits ripening. Conserved alternative splicing events did not appear to be stage-specific. Clustering fruit developmental stages across the four species based on alternative splicing profiles resulted in species-specific clustering, suggesting that diversification of alternative splicing contributes to lineage-specific evolution in fleshy fruits.

**Conclusions:**

We obtained high quality transcriptomes and alternative splicing events during fruit development across the four species. Dynamics and nonconserved alternative splicing were discovered. The candidate stage-specific AS genes involved in fruit ripening will provide valuable insight into the roles of alternative splicing during the developmental processes of fleshy fruits.

**Supplementary Information:**

The online version contains supplementary material available at 10.1186/s12864-021-08111-1.

## Background

Alternative splicing (AS) is an important co-transcriptional modification that greatly expands the diversity of the transcriptome and proteome in eukaryotes [1–3]. Approximately 95% of genes undergo AS in humans [4], and more than 60–85% of genes are alternative spliced in plants [5–8]. Moreover, alternative splicing is closely related to environmental stress and dynamically regulates plant growth [1, 9, 10]. In view of the universality and importance of AS, it has become a hotspot topic in the field of transcriptome.

AS has been proven to play important roles in a variety of plant processes, including fruit ripening [11–15]. In maize (*Zea mays* L.), researchers have found that missplicing of U12-type introns in *rgh3* can cause aberrant endosperm cell differentiation and proliferation [16]. In rice (*Oryza sativa*), OsPPR939 plays crucial roles in plant growth and pollen development by splicing introns 1, 2, and 3 of mitochondrial *nad5* [17]. Fruits can be divided into fleshy fruits and dry fruits according to the types of fruit walls [18]. Tomato (*Solanum lycopersicum*) is the model species for studying fleshy fruits [19]. The expression and alternative splicing of cuticular water permeability (*cwp*) are effected by low temperature, and *cwp* controls the development of cuticular microfissuring and subsequent fruit dehydration in tomato [20]. Although alternative splicing plays an important role in tomato fruit ripening, there are few studies on AS during fruit development in other fleshy fruits. In particular, many fleshy fruits are economically and nutritionally important species, and it is essential to analyse alternative splicing in fleshy fruits.

With the increase in high-quality RNA-Seq data, a comparison of AS among multiple tissues was carried out, and significantly different AS event profiles among ten tissues were found [21]. In addition, some studies of AS were performed at the multispecies level and reported that conserved AS generally indicate functional importance [22–24]. Based on *Populus* and *Eucalyptus*, 71 conserved AS events were found to be related to wood formation in the two species, in which AP2 plays a role in a variety of plant regulatory processes and Aux/IAA proteins are likely to be involved in the early response to auxin signalling [25]. In addition, 537 conserved AS events in 485 genes were identified between *Brassica* and *Arabidopsis*: for example, conserved skipping of two exons occurred in *PsbP,* which has a bipartite transit sequence containing the information for import across the chloroplast envelope as well as for targeting to the thylakoid, as well as alternative isoforms [26]. Nevertheless, little is known about the conservation of alternative splicing during fruit development among the fleshy fruits.

Here, we carried out a comparative analysis of alternative splicing during fruit development across cucumber, melon, papaya and peach spanning 123.2 million years [27] to explore the patterns and conservation of AS. We tried to answer the following questions: (1) what are the AS distributions at the two developmental stages across the four fleshy fruits, (2) what are the roles of AS during fleshy fruit development, and (3) what evolutionary characteristics appear across the four species?

## Results

### Transcriptomes diversity across four plants with fleshy fruits

We used RNA-Seq data for two fruit developmental stages (immature stage and ripe stage) from cucumber, melon, papaya, and peach, and each developmental stage had two biological replicates (Additional file 1: Table S1). The data were downloaded from the NCBI SRA database (Accession: PRJNA381300). In total, 4 ~ 12 million raw reads were obtained per sample, and then Trimmomatic [28] was applied to trim the adapter sequences and filter out the low-quality reads. Finally, over 70% of high-quality reads were remained for each sample (Table 1).

**Table 1:** RNA-Seq data from the four species

Species	Stage	Clean paired-end reads	Percentage of high-quality reads	Percentage of mapped reads
Cucumber	immature_rna-seq_1	3,661,481	90.92%	92.16%
	immature_rna-seq_2	3,342,782	75.52%	93.26%
	ripe_rna-seq_1	7,800,230	93.35%	87.80%
	ripe_rna-seq_2	6,471,011	77.37%	94.61%
Melon	immature_rna-seq_1	4,043,943	89.96%	90.27%
	immature_rna-seq_2	4,238,989	78.25%	94.00%
	ripe_rna-seq_1	11,761,127	92.21%	67.05%
	ripe_rna-seq_2	5,608,864	81.69%	94.89%
Papaya	immature_rna-seq_1	3,046,427	75.09%	83.45%
	immature_rna-seq_2	3,325,695	71.06%	89.43%
	ripe_rna-seq_1	4,053,807	83.04%	87.80%
	ripe_rna-seq_2	4,274,400	73.55%	92.14%
Peach	immature_rna-seq_1	3,298,825	90.65%	82.21%
	immature_rna-seq_2	5,807,465	73.90%	85.66%
	ripe_rna-seq_1	4,129,968	90.22%	81.59%
	ripe_rna-seq_2	6,417,825	75.65%	84.06%

To assemble the transcriptomes for the four species, the high-quality reads were mapped to the reference genomes using STAR [29]. On average, over 80% of reads were aligned to the genomes, except for the melon “ripe_rna-seq_1” sample (67.05%) (Table 1). In addition, the correlation analyses showed highly positive correlations between the biological repeats (Additional file 2: Fig. S1) and the sequencing depth for each sample was sufficient to perform alternative splicing analyses (Additional file 3: Fig. S2). Next, the mapped reads were assembled using StringTie [30]. For each species, transcript assemblies for the two stages were independently constructed, and a set of stringent criteria was applied to minimize the number of false, misassembled and poorly supported transcripts (see Methods, Additional file 4: Fig. S3). Then, the transcriptomes of the two developmental stages were merged. Finally, we obtained comprehensive transcriptomes for the four plants with fleshy fruits plants during fruit development (Table 2). Among the four species, the proportion of expressed genes ranged from 48.47% (14,532 genes in melon) to 58.08% (14,123 genes in cucumber) (Table 2). Meanwhile, we obtained a list of stage differentially expressed genes (Additional file 5: Table S2).

**Table 2:** Summary of high-quality transcriptomes

Species	Number of transcripts	Number of expressed genes	Percentage of expressed genes
Cucumber	20,720	14,123	58.08%
Melon	20,884	14,532	48.47%
Papaya	18,999	12,612	53.39%
Peach	22,902	15,363	57.17%

### Temporal alternative splicing dynamics during fleshy fruit development

Based on the above transcriptomes, the program SUPPA2 [31] was applied to identify intron retention (IR), exon skipping (ES), alternative 3′ splice site (A3SS), and alternative 5′ splice site (A5SS) events. A total of 4577, 4059, 3337 and 3198 events were identified (Additional file 6: Fig. S4), and 14.96–17.48% of multiexon genes exhibited alternative splicing (Additional file 7: Fig. S5). We also found that the retained introns were significantly shorter than other introns (Wilcoxon test, *p* − *value* ≤ 2.2*e* − 16) (Additional file 8: Fig. S6A), and there were positive correlations between the number of exons or introns and the ratio of ES genes or IR genes (Additional file 8: Fig. S6B, and Fig. S6C). These genic characteristics were consistent with a previous study [32].

AS is dynamically regulated during developmental processes in eukaryotes [33]. To characterize the dynamics of AS during fleshy fruit development, we compared the distribution of AS at the immature and ripe stages of cucumber, melon, papaya and peach. First, we mapped the reads of each sample to the transcriptome and then calculated the PSI (percent spliced in) [4] for each AS event. An AS event is considered to occur at one stage when the PSI of the AS event is more than or equal to 0.05 and less than or equal to 0.95.

**Fig. 1:**
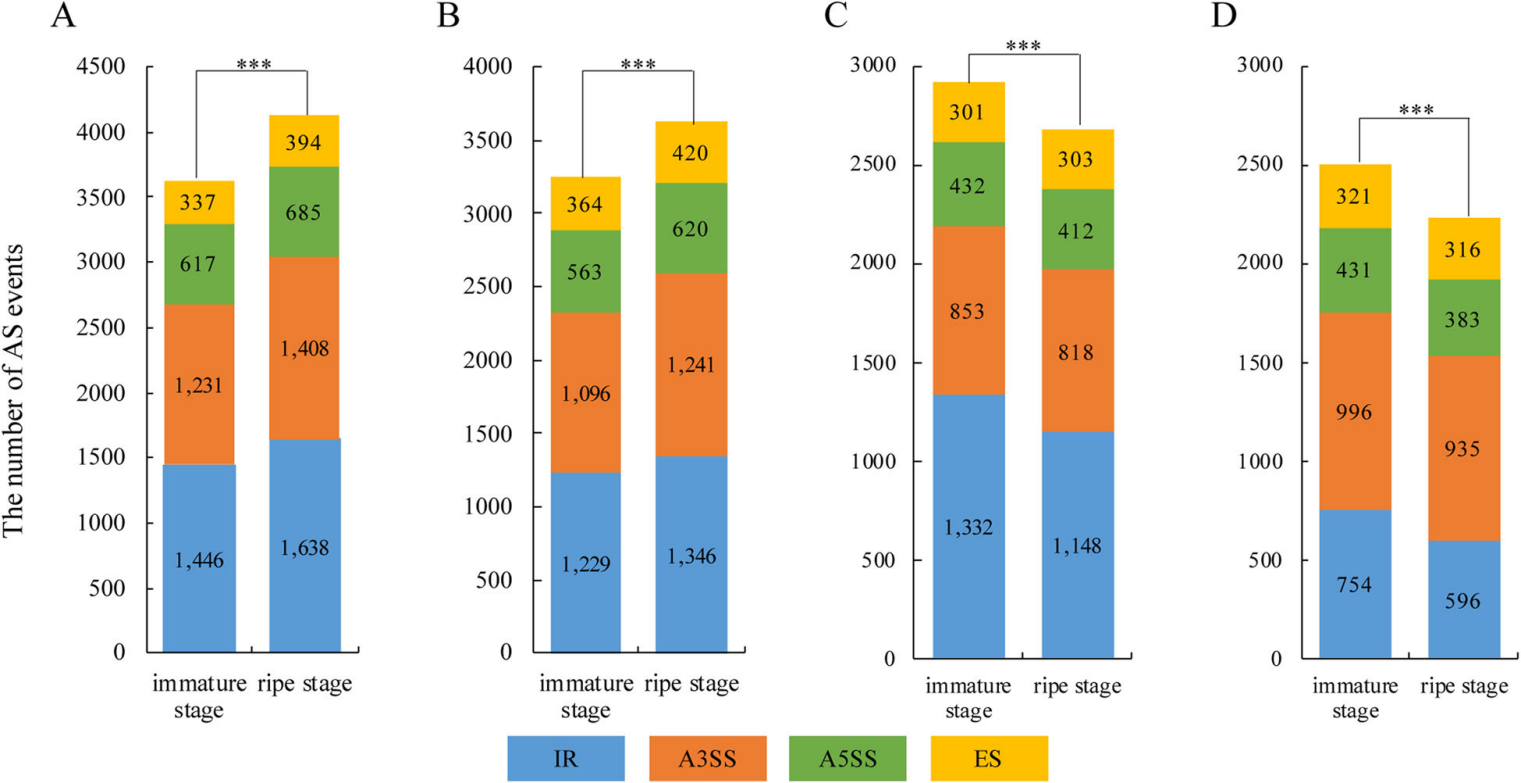
The alternative splicing distribution for the fruits at different stages. Fisher’s exact test was used to compare the number of AS events at different stages. The percent spliced in (PSI) of these AS events ranged from 0.05 to 0.95. Asterisks represent the significant difference. a cucumber. b melon. c papaya. d peach

As shown in Fig. 1, significantly more events (*PSI* ∈ [0.05,0.95]) occurred at the ripe stage than that at the immature stage in cucumber and melon (Fisher’s exact test, cucumber: *p-value* < 2.2e-16, melon: *p-value* < 2.2e-16), and the number of AS events increased with fruit development, as was also seen in soybean [6]. Nevertheless, the trend was opposite in papaya and peach (Fisher’s exact test, papaya: *p-value* < 2.2e-16, peach: *p-value* = 1.02e-14) (**Fig. 1**). In addition, we also found that the most and the least frequent AS types were IR and ES at the two developmental stages in fleshy fruits of cucumber, melon and papaya, which is consistent with studies in soybean, maize, and *Arabidopsis* [6, 34–37]. Nevertheless, in peach fruits, there were more A3SS events than IR events (Fig. 1). Thus, peach fruits displayed a different pattern of AS. A previous study in peach had discovered that IR (2.76%) was the rarest AS event; conversely, ES was the most prevalent event [38]. In addition, we obtained a list of stage differentially alternatively spliced genes (Additional file 9: Table S3).

### Genes involved in fruit ripening undergo stage-specific alternative splicing

**Fig. 2:**
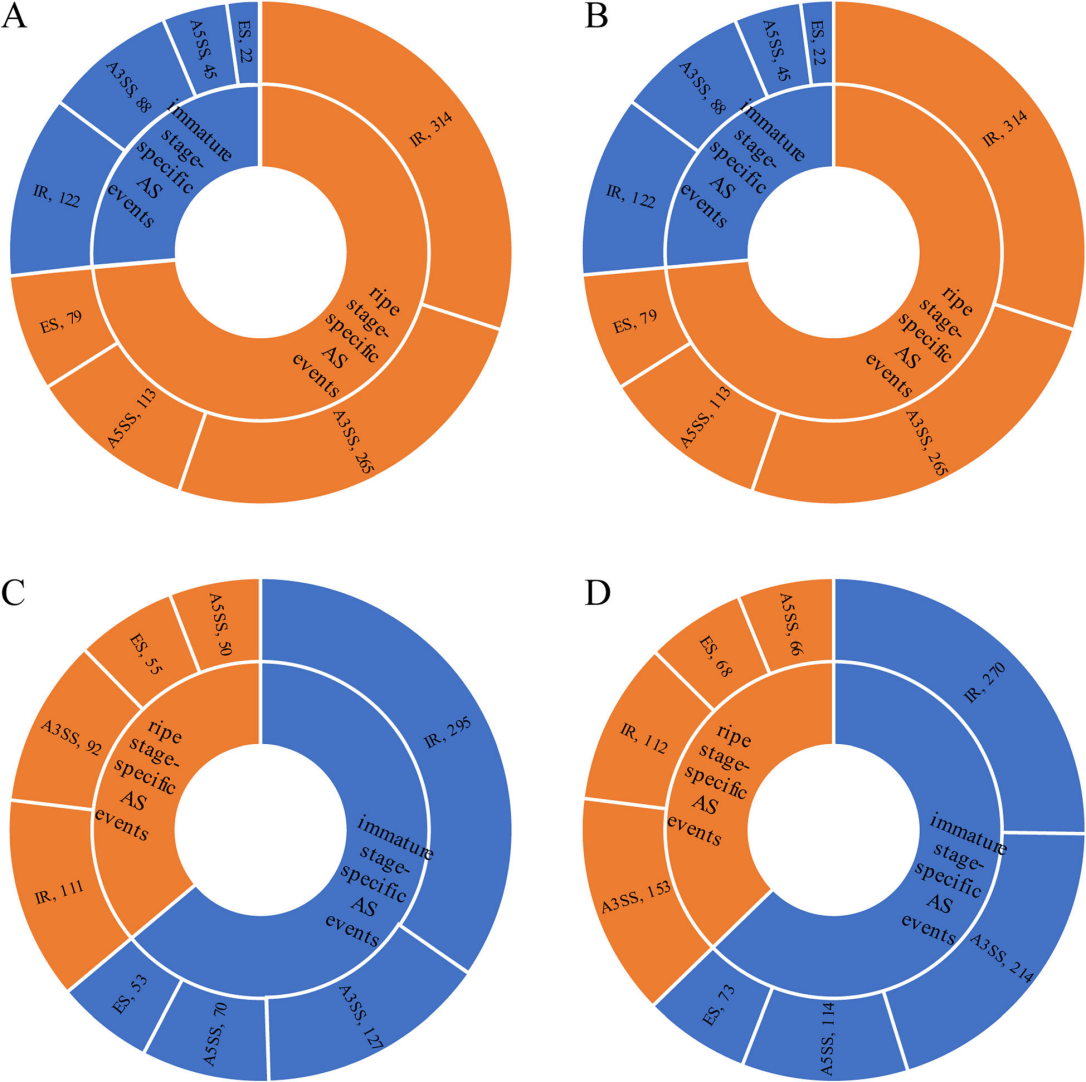
Distribution of stage-specific AS events. a cucumber. b melon. c papaya. d peach

Previous studies have reported that stage-specific AS regulates specific developmental processes in eukaryotes [14, 34, 39]. Therefore, we wanted to identify the stage-specific AS events at two stages during fruit development in the four species. An AS event was considered a stage-specific event if it only occurred at one stage (*PSI* ∈ [0.05,0.95]). More ripe stage-specific AS events than immature stage-specific AS events were found in cucumber and melon; in contrast, more immature-specific AS events than ripe-specific AS events were observed in papaya and peach (Fig. 2). These results indicated the more AS events and the more stage-specific AS events.

We further tried to investigate whether the genes related to the fleshy fruit ripening process undergo stage-specific AS events. We considered that these genes might regulate fruit ripening by stage-specific AS. Thus, the genes with stage-specific AS were aligned against the genes involved in tomato fruit ripening [40] using BLASTP (version: 2.8.1) [41], and the corresponding ITAG4.1 (ftp://ftp.solgenomics.net/tomato_genome/annotation) annotation was used [42]. As shown in Table 3, some genes related to the process of fleshy fruit ripening experienced stage-specific AS (the detailed mapped Gene IDs are listed in Additional file 10: Table S4). Interestingly, the genes participating in ethylene biosynthesis and signalling underwent stage-specific AS during the whole process of fruit development in the four species. Moreover, there were more genes with ripe stage-specific AS events associated with cell wall structure, reflecting the process of fruit softening.

**Table 3:** Genes involved in stage-specific AS and related to fleshy fruit ripening

Species	Pathway	Genes with immature stage-specific AS	Genes with ripe stage-specific AS
Cucumber	Carotenoid	*PSY1*	*PDS, ZISO*
	Cell wall structure	*SlXTH3, SlXTH2*	*SlXTH3, SlXTH3, teg1A/TBG3, SlXTH2, PG2a, TBG4, TBG4*
	Ethylene biosynthesis and signalling	*LeCTR1, LeETR5*	*LeACS2, LeACS4, SlTPR1, LeACS1A, LeCTR1*
	Flavonoid/anthocyanin		*4CL, 4CL-like*
	Transcription factor		*AP2a*
Melon	Carotenoid		*CrtISO*
	Cell wall structure		*teg1A/TBG3, PG2a, TBG4*
	Ethylene biosynthesis and signalling	*LeCTR1, LeETR5*	*LeACS2, LeACS4, LeETR4, SlTPR1, LeETR2, LeACS1A, LeETR3/NR, LeETR6, LeCTR1, LeETR5, LeETR1*
	Flavonoid/anthocyanin		*LoxC*
	Transcription factor	*CNR*	*AP2a*
Papaya	Carotenoid	*CrtISO*	*PTOX*
	Cell wall structure	*PG2a*	*teg1A/TBG3, LeEXP1, PG2a, TBG4*
	Ethylene biosynthesis and signalling	*LeETR4, LeEIL4, SlTPR1, LeETR2, LeETR3/NR, LeETR6, LeCTR1, LeETR5, LeETR1*	*LeACS2, LeACS4, LeACO5, LeACO1, E8, LeCTR1*
	Flavonoid/anthocyanin	*4CL, 4CL-like*	*4CL, 4CL-like*
	Transcription factor		*AP2a, FUL2, FUL1, NOR, NAC4*
Peach	Carotenoid	*ZEP, CrtISO*	
	Cell wall structure	*PG2a*	*teg1A/TBG3, TBG4*
	Ethylene biosynthesis and signalling	*LeACO5, LeACO1, E8, LeCTR1*	*SlTPR1, LeCTR1, LeETR5*
	Flavonoid/anthocyanin	*4CL, 4CL-like*	
	Transcription factor	*AP2a, FUL2, FUL1, NOR, NAC4*	*AP2a, FUL2, FUL1, NOR, NAC4*

### Low conservation and species-specific clustering patterns of alternative splicing in fleshy fruits

We wanted to determine whether there were conserved AS events during fruit development across the four species. Thus, we identified junction-based conserved AS events among the four species (see Methods). In total, we detected 2001 conserved AS events among the four fruits (Table 4), and each event was present in two or more orthologous genes. As displayed in Table 4, A3SS was the most common type of conserved AS and occupied of 41.48%, IR was the second most common type of conserved AS and occupied 32.43%, and ES was the least common type of conserved AS and occupied 9.60%. Previous studies have reported that A3SS or IR is the significantly overrepresented conserved events between species [25, 26] or among species [32, 43].

**Table 4:** Conserved AS events

Species	IR	A3SS	A5SS	ES	All
Cucumber	299	363	144	74	880 (43.98%)
Melon	264	348	142	75	829 (41.43%)
Papaya	51	50	18	19	138 (6.90%)
Peach	35	69	26	24	154 (7.69%)
All	649 (32.43%)	830 (41.48%)	330 (16.49%)	192 (9.60%)	2001

“Percentage” is based on the total number of conserved AS events.

**Fig. 3:**
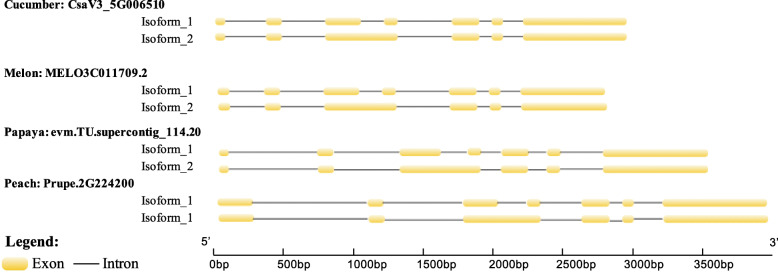
SR genes exhibiting a highly conserved IR event (the third intron was retained) in the four species

**Fig. 4:**
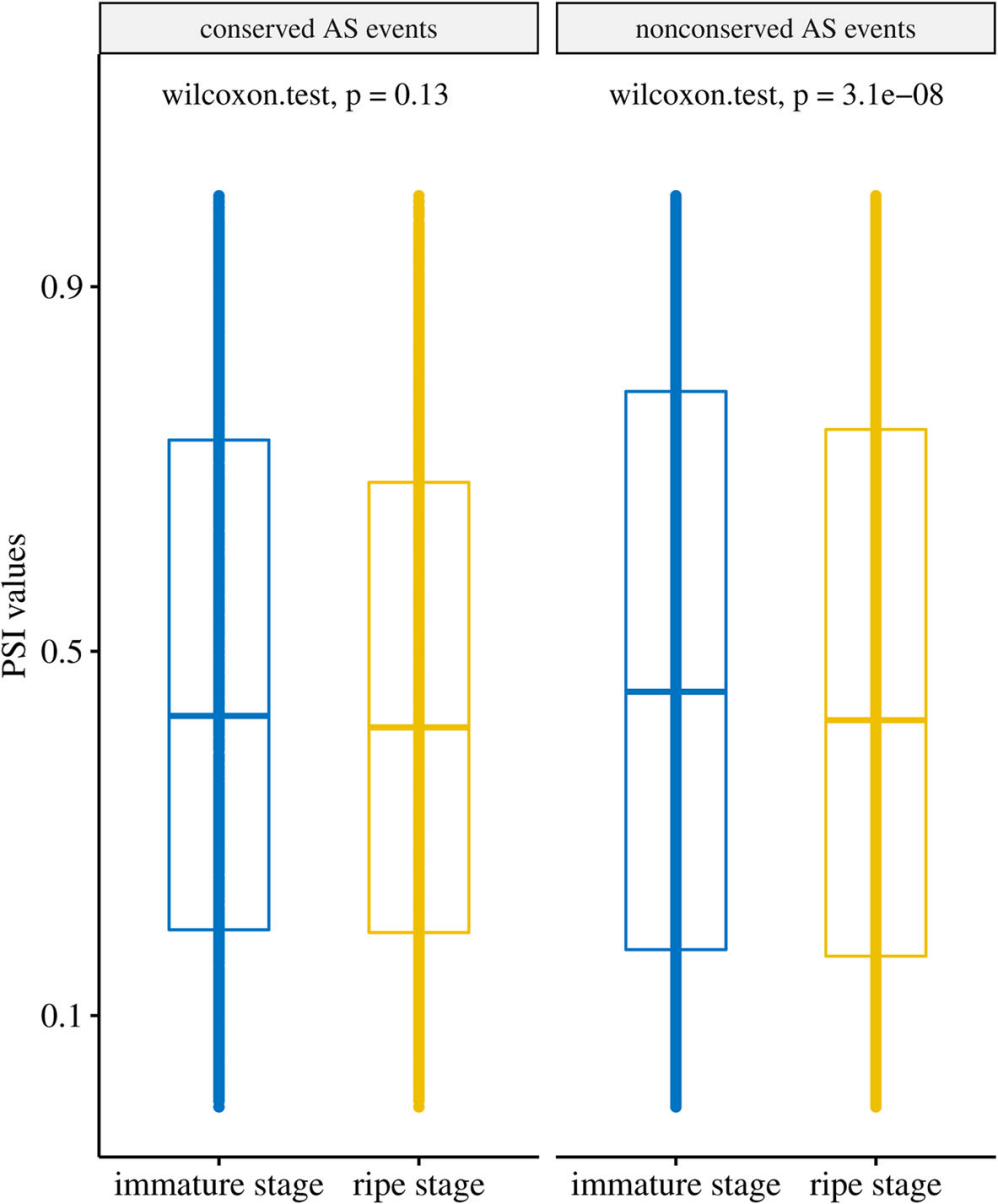
The distribution of PSI values of conserved and nonconserved AS events at immature- and ripe-stages. *P-values* were calculated by Wilcoxon test

In addition, we also found that 13 AS events were conserved among the four species. These events were defined as highly conserved AS events (Table 5**,** Additional file 11: Table S5). Among these AS events, the genes encoding serine/arginine-rich splicing factor [44] experienced a highly conserved IR event between the third and fourth exons across the four species during fruit development (Fig. 3). A previous study also revealed that the AS events of SR genes were conserved in other tissues of other species [43]. We further speculate that conserved AS events may occur without stage bias. Therefore, we investigated the PSI values of conserved AS events and observed that there was no significant difference in the PSI values between the immature stage and ripe stage for conserved AS events (Wilcoxon test, *p-value = 0.13*). In contrast, a significant difference in the PSI values between the immature stage and ripe stage for nonconserved AS events was observed (Wilcoxon test, *p-value = 3.1e-08*) (Fig. 4). The result indicated that conserved AS events may occur without stage bias during fruit development.

**Table 5:** Highly conserved AS events and the corresponding genes

Type	Gene IDs	Gene description
A3SS	CsaV3_6G042440, MELO3C007487.2, evm. TU.supercontig_196.5, Prupe.6G174000	Transmembrane protein
A3SS	CsaV3_1G038040, MELO3C015241.2, evm. TU.supercontig_48.51, Prupe.6G304400	G patch domain-containing protein 8; D111/G-patch domain-containing protein
ES	CsaV3_5G030480, MELO3C011886.2, evm. TU.supercontig_43.33, Prupe.1G232700	cyclin-dependent kinase G-2-like isoform X1; Protein kinase superfamily protein
A3SS	CsaV3_7G030160, MELO3C015907.2, evm. TU.supercontig_18.90, Prupe.3G007400	ATP-dependent RNA helicase, putative; RNA helicase family protein
IR	CsaV3_5G006510, MELO3C011709.2, evm. TU.supercontig_114.20, Prupe.2G224200	Arginine/serine-rich splicing factor, putative; RSZ32 RNA-binding (RRM/RBD/RNP motifs) family protein with retrovirus zinc finger-like domain
A3SS	CsaV3_1G039610, MELO3C015383.2, evm. TU.contig_46341, Prupe.6G337000	kinesin-like protein KIFC3; P-loop containing nucleoside triphosphate hydrolases superfamily protein
IR	CsaV3_1G038040, MELO3C015241.2, evm. TU.supercontig_48.51, Prupe.6G304400	G patch domain-containing protein 8; D111/G-patch domain-containing protein
A3SS	CsaV3_4G009190, MELO3C022635.2, evm. TU.supercontig_14.172, Prupe.3G255200	protein RIK isoform X2, RS2-interacting KH protein
A3SS	CsaV3_7G031250, MELO3C023531.2, evm. TU.supercontig_234.14, Prupe.2G009600	SURP and G-patch domain-containing protein 1-like protein; SWAP (Suppressor-of-White-APricot)/surp domain-containing protein/D111/G-patch domain-containing protein
ES	CsaV3_6G047540, MELO3C007946.2, evm. TU.supercontig_6.317, Prupe.7G175500	serine/arginine-rich splicing factor SR45a; RNA-binding (RRM/RBD/RNP motifs) family protein
A3SS	CsaV3_5G004040, MELO3C005554.2, evm. TU.supercontig_1025.1, Prupe.5G073700	Oxysterol-binding protein; OSBP (oxysterol binding protein)-related protein 2A
A3SS	CsaV3_3G042630, MELO3C009973.2, evm. TU.supercontig_470.3, Prupe.3G287500	ATP-dependent RNA helicase; DEAD box RNA helicase family protein
ES	CsaV3_4G007970, MELO3C016139.2, evm. TU.contig_29215, Prupe.4G154700	Mitogen-activated protein kinase; MAP kinase 19

“Gene IDs”: the gene id in cucumber, melon, papaya, and peach.

**Fig. 5:**
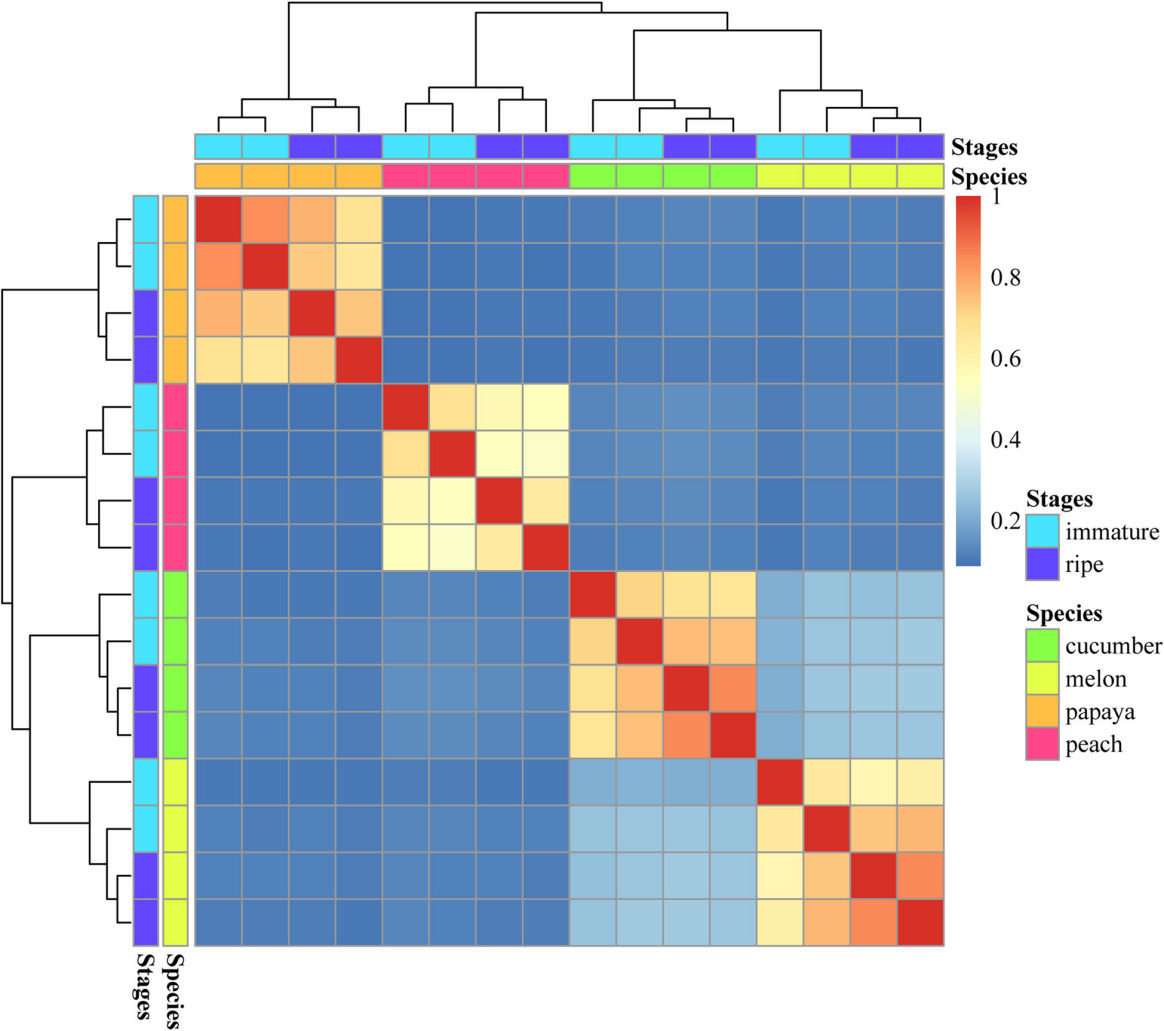
Clustering of alternative splicing profiles among the fruits. Clustering was based on the presence and absence of AS events in the 8865 orthologous genes among the four species. The colour code above the heatmap represents species and stages

Changes in AS represent a major source of species-specific differences [45, 46]. Therefore, we investigated whether a similar pattern could be seen during fruit development in the four plants. We compared AS profiles (see Methods) across different developmental stages and species. By clustering the presence/absence of AS profiles for the samples based on the one-to-one orthologous genes, we observed a species-specific AS clustering pattern across the four plants (Fig. 5). Further, we got a list of genes that undergo species-specific alternative splicing (Additional file 12: Table S6). This indicated that ‘switch-like’ regulation of alternative splicing might also be a major source of species-specific differences in plants.

## Discussion

Consistent with previous studies, we found that AS significantly increased transcriptome diversity in plants with fleshy fruits, and approximately 20% of genes were alternatively spliced. We compared the alternative splicing between immature and ripe stages across the four fleshy fruits and revealed the dynamics and low conservation of AS within and among fleshy fruits. IR was not always the most common event, and the number of AS events was significantly different between the ripe stage and the immature stage. Some genes involved in fruit ripening underwent stage-specific AS. Conservative AS is not shown stage-specific. The clustering of fruit developmental stages across the four species based on AS profiles displayed species-specific clustering.

**Fig. 6:**
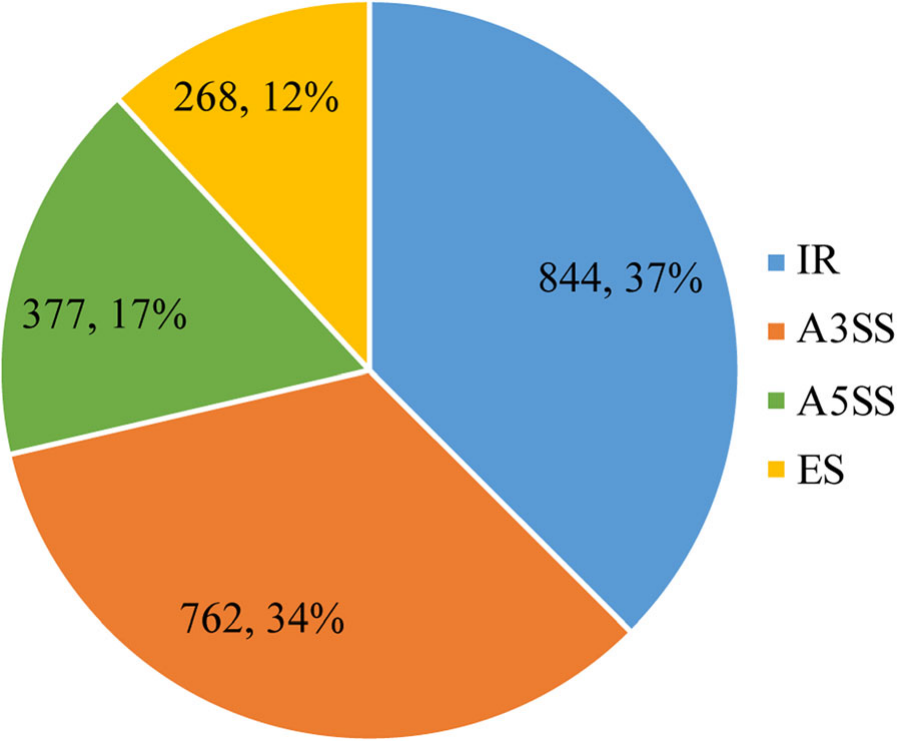
The distribution of alternative spliced events in leaves from peach

In our analysis, illumina paired-end data was used to discover the AS events, so we firstly assembled the transcripts. To avoid assembly errors, a set of stringent criteria was applied to validate the transcripts. We obtained high-quality and nonredundant transcriptomes during the fruit developmental process for fleshy fruits. Here, 14.96% (1726 in papaya)-17.48% (2269 in cucumber) of multiexon genes were alternatively spliced. In cucumber, melon and papaya, IR was the most prevalent, which is commensurate with the results observed in soybean, maize and *Arabidopsis* [6, 34–37]. However, the distribution of AS in peach contradicts the findings of previous studies. The most common AS was A3SS, which accounted for 39% of all the observed AS events, suggesting that the prevalent AS type differs among fleshy fruits. To examine whether A3SS is the most common AS in other tissues in peach, we followed the same process to assemble the transcriptome and identify AS events of leaves in peach (Additional file 13: Table S7). We found that IR was the most common AS event (844 events occupying 37%) (Fig. 6), indicating that the distribution of AS is related to the tissues in peach. We also observed that significantly more events occurred at the ripe stage than that at the immature stage in cucumber and melon, while the pattern was opposite in peach and papaya. On the one hand, only two developmental stages were investigated. On the other hand, only four fleshy fruits were investigated. Therefore, with more developmental stages and more species, we will further confirm whether the pattern is general or specific.

The ripening of fleshy fruits is a complex developmental process regulated by many biochemical processes, and many genes are associated with this process [47]. In our study, we found that some genes related to fruit ripening underwent stage-specific AS. For the genes participating in the ethylene biosynthesis and signalling pathway, stage-specific alternative splicing occurred at both the immature and ripe stages, such as for *ETR* and *ACO*. Additionally, cell wall softening plays an important role in the textural changes in fleshy fruits [48], and the genes involved in cell wall structure were found to have more ripe-specific AS than immature-specific AS; for example, *PG* and *TBG4*, which are essential for fruit softening [40, 49, 50], were identified. These results emphasize the roles of AS during fleshy fruit development.

Conserved AS events may play significant roles in multiple physiological processes in plants [51], and conserved AS generally indicates the functional significance [22–24]. Our results indicated that AS was not conservative and that its conservation was decided by the evolutionary distance. However, it was interesting that we did find conserved AS events, even though AS didn’t significantly change between the two stages. We think that it is possible that these conserved AS events may be regulated and change the proportion of alternative transcripts in a conserved way in these different plant species under different conditions.

Limited by the availability of RNA-Seq data, we only compared the immature and ripe stages of the four plants with fleshy fruits. The study of more temporally restricted samples during fruit ripening in more species with fleshy fruits will allow deeper exploration of the roles of AS during fruit development and discovery of differences and similarities during fruit development among species. Although a set of stringent criteria was applied to avoid assembly errors, the long-read sequencing strategy would simplify the process and reduce the errors of assembly. We hope to carry out further analysis in the future.

## Conclusions

In summary, based on the RNA-Seq data at immature and ripe stages from the fleshy fruits of cucumber, melon, papaya and peach, we obtained high-quality transcriptomes and AS events. The collection of AS events indicated that IR was not necessarily the most common AS in fleshy fruits. Then, in comparisons within species, we found that these fruits have different AS strategies at the immature and ripe stages. Among the species, low conservation and species-specific AS patterns were observed. Moreover, we observed that some genes related to fleshy fruit ripening undergo stage-specific AS during fleshy fruit development. These stage-specific AS events will provide an experimental candidate dataset for the mechanism of AS in fleshy fruit ripening. These results will help us understand the important roles of AS during fleshy fruit development.

## Methods

### Data sources

All analyses were performed on public data. The raw RNA-Seq data come from the NCBI BioProject, accession code PRJNA381300 [52], and data were downloaded from the NCBI Sequence Read Archive (SRA) database under accession code SRP078156: we collected cucumber (*Cucumis sativus* L.), melon (*Cucumis melo* L.), papaya (*Carica Papaya* L.) and peach (*Prunus persica* L.) RNA-Seq datasets. Cucumber at 9 days post anthesis (DPA) was described as immature green stage, and at 27–30 DPA was described as fully ripe stage [53], therefore, for cucumber, we chose the RNA-Seq data from 10 and 30 DPA as the immature and ripe stage, respectively. The cucumber samples used were grown under standard greenhouse condition. When melon reaches 10 DPA, its ovarian epidermal cells stop proliferating leading to young fruit stage, and when comes to 30 DPA, melon cell expansion stops with maximum fruit volume and flesh firmness [54]. Thus, for melon, we also chose RNA-Seq data from 10 and 30 DPA as the immature and ripe stages, respectively. The melon samples used were grown under standard greenhouse condition. For peach, we directly downloaded RNA-Seq from immature and ripe fruit flesh tissues. The peach samples used were grown under orchards in the Zhengzhou Fruit Research Institute, Chinese Academy of Agriculture Sciences 16 (Zhengzhou, China). For papaya, they sequenced four development stages from immature to fully ripen. Among these stages, we chose stage 1 and stage 4 as the immature and ripe stages, respectively. The papaya samples used were grown under a plantation associated with South China Agricultural University in Guangzhou, China. All of the plants were grown in normal conditions, thus they are comparable. In addition, there were two replicates for each condition (Additional file 1: Table S1). All these sequences were 150 bp paired-end sequencing reads (Table 1).

### Data processing and transcriptome assemblies

Adaptors and low-quality reads were trimmed using Trmmomatic (version:0.36) [28] with the parameters “SLIDINGWINDOW: 4:15” and “MINLEN: 36”. The trimmed paired reads were mapped to the reference genome (Additional file 14: Table S8) using STAR (version: 2.6.1) [29] with --twopassMode Basic, −-outSAMstrandField intronMotif, −-outFilterIntronMotifs, RemoveNoncanonical, −-outFilterMismatchNmax 2, −-outSAMprimaryFlag AllbestScore, and --outSAMattrIHstart 0 parameters, and to allow more junction reads to map to the novel junctions, the 2-pass mapping mode (−-twopassMode Basic) was used. Then, a bam file was generated for each sample.

Based on these alignments, transcripts were assembled using StringTie (version: 1.2.3) [30] with the default parameters for each sample. Then, the transcripts obtained from replicates were merged with TACO (version: 0.7.3) [55]. Thus, for each fleshy fruit, we obtained two transcriptomes from the immature and ripe stages.

To obtain a high-quality reference transcriptome of fruit development for each species, a series of quality filters were applied for the transcriptome of each stage. First, transcripts that were poorly supported by junction reads were removed: for each novel splicing junction, there were fewer than 4 reads supported in both replicates or fewer than 10 reads supported in one replicate [56]. These transcripts were retained, and the expression level in the two replicates were both greater than 1 TPM (transcripts per million). Moreover, to detect transcripts from annotated genes or novel genes, GffCompare (version: 0.9.8) [57] was applied between the reference annotation and assembled transcripts, and transcripts that were classified as novel genes and antisense genes (class code equal to “u”, “x”, and “s”) were removed [35]. In addition, to avoid assembly error, we removed the transcripts with single exons that were not equal to the reference annotation. Finally, the high-quality transcripts from two developmental stages were merged using an in-house Perl script: for the transcripts from different stages with the same intron coordinates but different transcript lengths, we kept the longer one. Based on the above, the reference transcriptomes of fruit development were obtained, and they were used as reference transcriptomes for downstream analysis.

### Identification of stage differentially expressed genes

We used r-package DESeq2 (version: 1.30.1) [58] for differential expression. Based on the samples’ clean RNA-Seq data, the expression values of genes were quantified by Salmon [59]. Using the expression matrix of every fruit generated by Salmon (version: 0.13.0), DESeq2 was used to calculate differential expression levels between two stages for every fruit. We further identified genes which adjust *p*-value < 0.05 and |log2FoldChange| > 1 as differential expressed gene.

### Identification of AS events and stage differentially alternatively spliced events

For each fleshy fruit, SUPPA2 [31] was employed to annotate AS events. We analyzed only the four main types of AS events: IR, ES, A5SS, and A3SS. Then, based on the samples’ clean RNA-Seq data, the expression values of referenced transcripts were quantified by Salmon (version: 0.13.0) [59], and according to the expression values of transcripts, SUPPA2 calculated the PSI values [4] for each AS event at different developmental stages: the AS events occurred when the *PSI* ∈ [0.05,0.95]. For differential AS events, we used script ‘diffSplice’ in SUPPA2 to calculate. Using AS event file (.ioe), PSI values file (.psi) and transcript expression file as input files, the results were generated with *p*-values and ΔPSI between stages for every event. When an AS event ‘s p-value < 0.05 and |ΔPSI| > 0.1, we identified this AS event as a differential alternatively spliced event. Genes with differentially alternatively spliced event could be further defined from those events.

### Identification of orthogroups and orthologues across the four species

OrthoFinder (version: 2.3.1) [60] was applied to group orthogroups with default parameters. Then, to obtain the one-to-one orthologous relationships, we performed the following steps. First, the single copy orthogroups generated by OrthoFinder were retained. Then, to obtain one-to-one orthologous genes from the many-to-many and one-to-many orthologues, we implemented BLASTP with an E-value cut-off of 1e-10. In addition, based on the results of BLASTP synteny blocks across four species were determined using MCScanX [61] with default parameters. Syntenic gene lists across four species were used for downstream analysis. Finally, the orthologues among the four species contained single copy orthogroups and the syntenic genes in the one-to-many or many-to-many orthogroups.

### Detection of conserved AS events

**Fig. 7:**
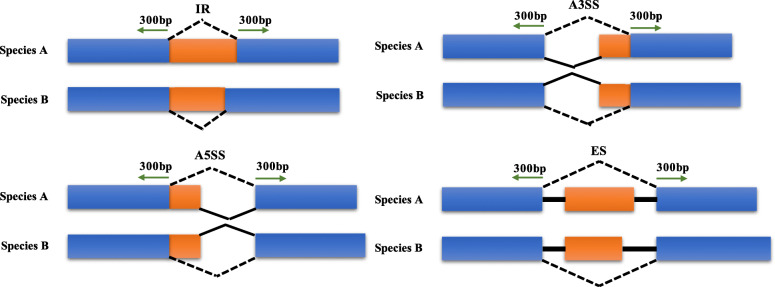
The identification of conserved alternative splicing events. Thirty- to 300-bp flanking exon sequences at alternative splicing junctions were extracted, and two AS events were considered conserved when the paired genes belonged to the same orthogroup and the paired flanking sequences of an AS were similar to another pair of flanking sequences

AS was independently annotated in each species. To detect conserved AS events across the four fleshy fruits, a junction-based method was applied [62]. First, we separately extracted the 30–300 bp flanking exon sequences for each alternative spliced junction; thus, an AS event could be represented by a pair of flanking sequences. Next, the flanking sequences were grouped into four datasets according to the AS types (IR, A3SS, A5SS, and ES). Each flanking sequence in a dataset was searched against all other flanking sequences of the same dataset with ab-tblastx in AB-BLAST (https://blast.advbiocomp.com, version: 3.0) (Fig. 7). After that, two AS events were considered as conserved when the AS genes belonged to the same orthogroup and the paired flanking sequences of an AS were similar to another pair of flanking sequences.

### Cluster analysis of AS profiles

To compare AS profiles across developmental stages and species, we constructed the presence/absence of AS profiles for each sample. We constructed a vector P = ({*N*_*i*_}), where *N*_*i*_ (i∈{1,..,8865}) was 1 or 0, which represented whether AS occurred or did not occur for the i-th orthologue in this sample; therefore, we obtained 16 presence/absence of AS profiles. Then, the binary distance was calculated for the presence/absence AS profiles [63]. Genes that did not undergo AS in all samples were excluded.

### Statistical tests

Fisher’s exact test was used for the comparison of AS distribution. In addition, for the conserved and nonconserved AS event datasets, the differences between the PSI of the immature and ripe stages were evaluated using the Wilcoxon rank sum test. All statistical tests were performed using the R statistical package.

## Supplementary Information

**Additional file 1: Table S1.** Sample information.

**Additional file 2: Fig. S1.** Gene expression correlations. Gene expression correlations were calculated using the Pearson correlativity method. a cucumber. b melon. c papaya. d peach.

**Additional file 3: Fig. S2.** Saturation analysis of junction detection. a-d Junction saturation results of cucumber samples. e-h Junction saturation results of melon samples. i-l Junction saturation results of papaya samples. m-p Junction saturation results of peach samples.

**Additional file 4: Fig. S3.** Pipeline of the construction of high-quality transcriptomes. Transcripts poorly supported by spliced junctions, transcripts with low abundance, antisense transcripts, transcripts from unknown genes and single exon transcripts not found in the reference were removed. a cucumber. b melon. c papaya. d peach.

**Additional file 5: Table S2.** Differentially expressed genes between two stages.

**Additional file 6: Fig. S4.** Distribution of AS events during fruit development.

**Additional file 7: Fig. S5.** Distribution of multiexon genes under alternative splicing.

**Additional file 8: Fig. S6** Relationship between the genic features and the alternative splicing (AS). a Comparison of the length of introns between retained introns and other introns. b Correlations between the number of exons and the ratio of ES genes. c Correlations between the number of introns and the ratio of IR genes.

**Additional file 9: Table S3.** Stage differentially alternatively spliced genes.

**Additional file 10: Table S4.** Genes involved in fruit ripening with stage-specific alternative splicing.

**Additional file 11: Table S5.** Highly conserved AS events and the corresponding genes.

**Additional file 12: Table S6.** Species-specific AS genes.

**Additional file 13: Table S7.** Statistics of transcriptomes from peach leaves.

**Additional file 14: Table S8.** Information on genome annotation.

## Data Availability

The genome and genome transfer format (GTF) file of *Cucumis sativus* L. and *Cucumis melo* L. were downloaded from the CuGenDB (http://cucurbitgenomics.org). The genome and genome transfer format (GTF) file of *Carica Papaya* L. and *Prunus persica* L. were downloaded from the Phytozome (https://phytozome-next.jgi.doe.gov). The raw RNA-Seq data were downloaded from the NCBI Sequence Read Archive (SRA) database (https://www.ncbi.nlm.nih.gov/sra) under accession code SRP078156. All data that support this study are included within the article and its additional files 1, 2, 3, 4, 5, 6, 7, 8, 9, 10, 11, 12, 13 and 14.

## Abbreviations

AS: Alternative splicing
*cwp*: Cuticular water permeability
PSI: Percent spliced in
TPM: Transcripts per million
IR: Intron retention
ES: Exon skipping
A3SS: Alternative 3′ splice sites
A5SS: Alternative 5′ splice sites
DPA: Days post anthesis
