# Supplementary figures and images for: Alternative splicing during fruit development among fleshy fruits
Source: BMC Genomics. 2021 Oct 26;22:762. doi: 10.1186/s12864-021-08111-1 (PMC8547070; doi:10.1186/s12864-021-08111-1)

A

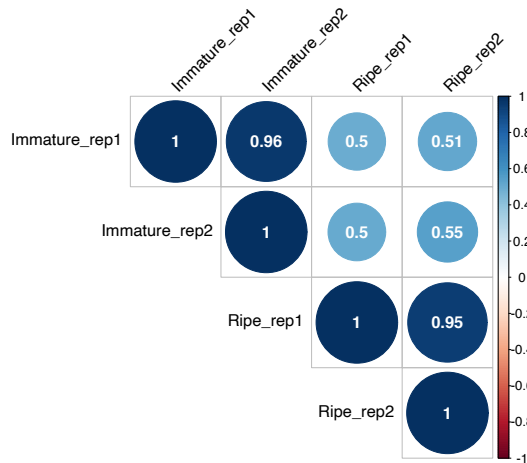

B

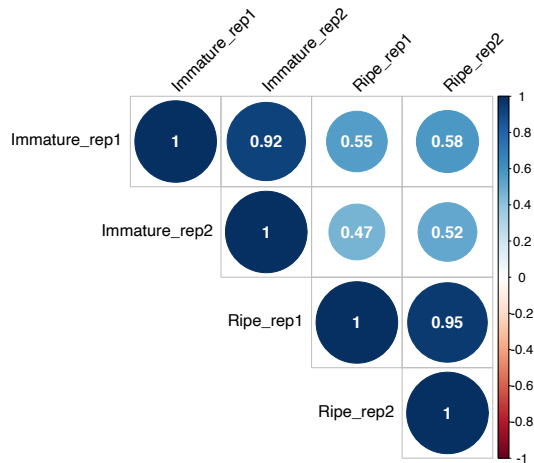

C

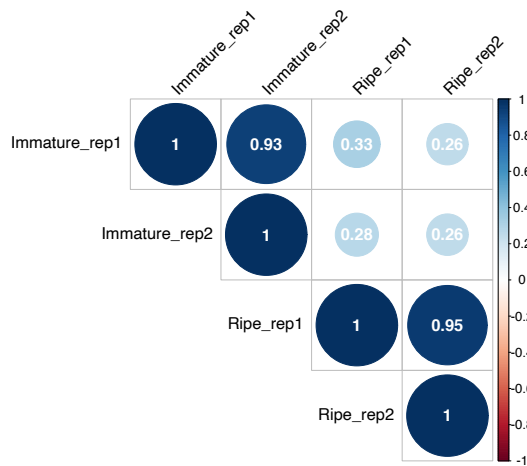

D

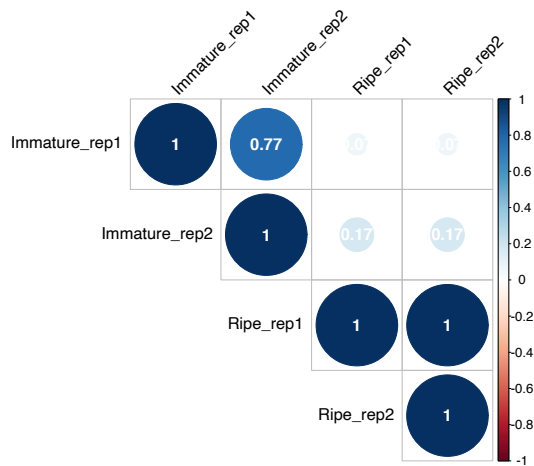

Supplement: Supplementary file 2 — Additional file 2: Fig. S1. Gene expression correlations. Gene expression correlations were calculated using the Pearson correlativity method. a cucumber. b melon. c papaya. d peach. [file 12864_2021_8111_MOESM2_ESM.pdf]

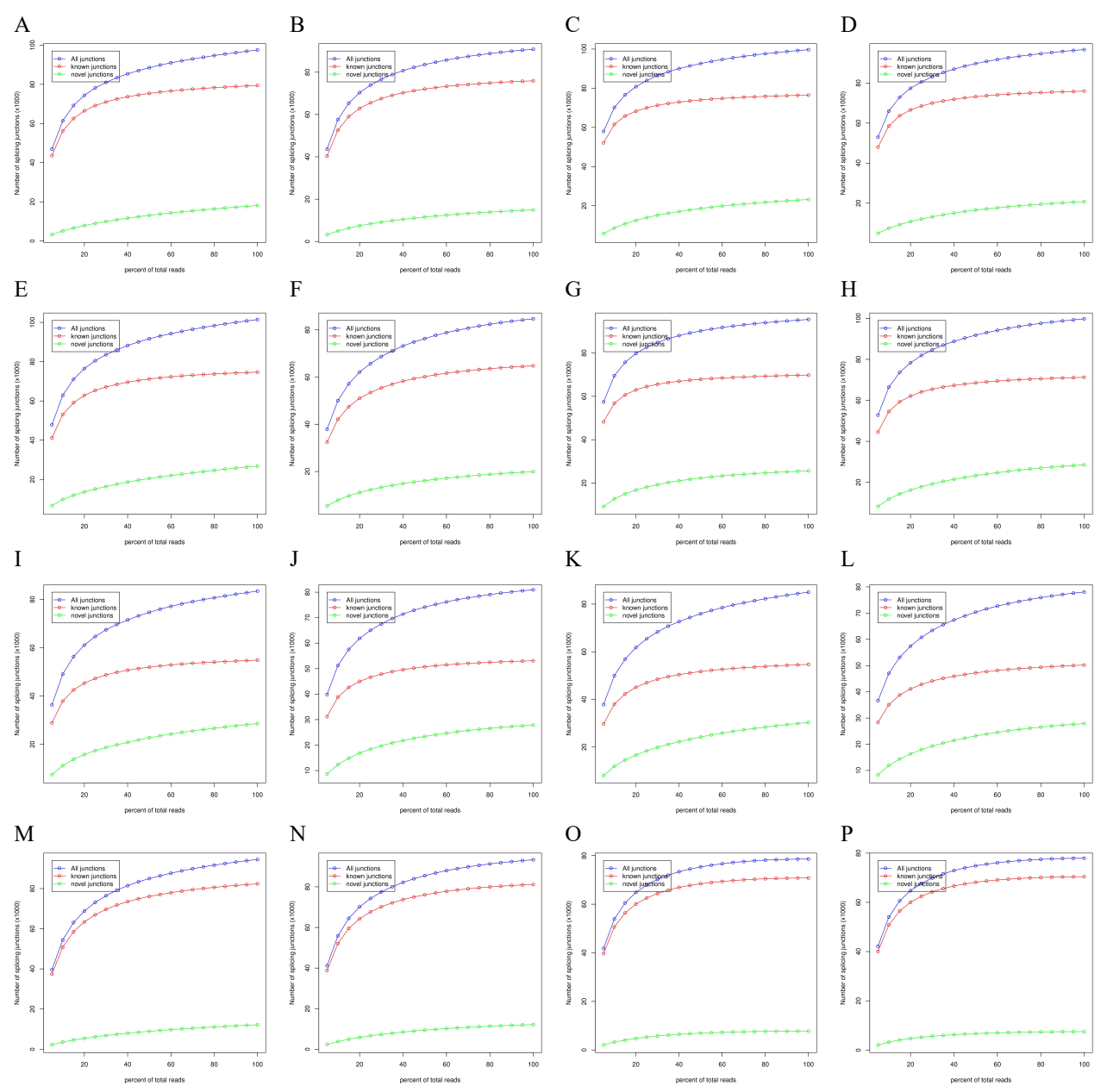

Supplement: Supplementary file 3 — Additional file 3: Fig. S2. Saturation analysis of junction detection. a-d Junction saturation results of cucumber samples. e-h Junction saturation results of melon samples. i-l Junction saturation results of papaya samples. m-p Junction saturation results of peach samples. [file 12864_2021_8111_MOESM3_ESM.pdf]

A

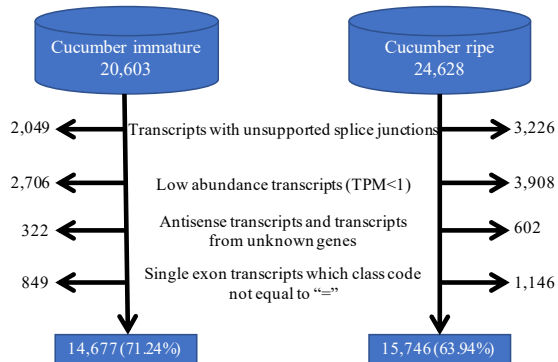

B

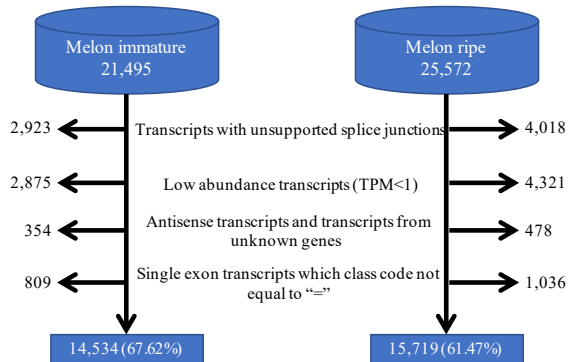

C

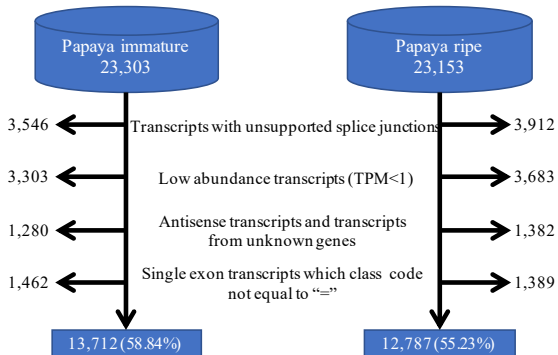

D

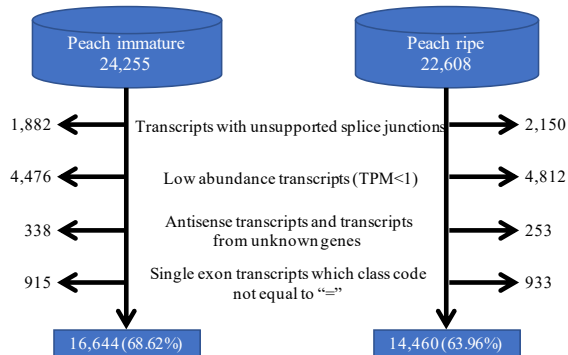

Supplement: Supplementary file 4 — Additional file 4: Fig. S3. Pipeline of the construction of high-quality transcriptomes. Transcripts poorly supported by spliced junctions, transcripts with low abundance, antisense transcripts, transcripts from unknown genes and single exon transcripts not found in the reference were removed. a cucumber. b melon. c papaya. d peach. [file 12864_2021_8111_MOESM4_ESM.pdf]

|          | IR    | A3SS  | A5SS | ES  | ALL   | Patterns                                                                            |      |
|----------|-------|-------|------|-----|-------|-------------------------------------------------------------------------------------|------|
| Cucumber | 1,850 | 1,545 | 756  | 426 | 4,577 | 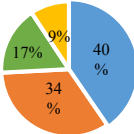  |      |
| Melon    | 1,567 | 1,361 | 677  | 454 | 4,059 | 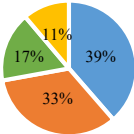 | IR   |
| Papaya   | 1,507 | 973   | 492  | 365 | 3,337 | 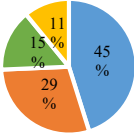 | A3SS |
| Peach    | 961   | 1,250 | 544  | 443 | 3,198 | 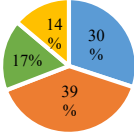 | A5SS |
|          |       |       |      |     |       |                                                                                     | ES   |

Supplement: Supplementary file 6 — Additional file 6: Fig. S4. Distribution of AS events during fruit development. [file 12864_2021_8111_MOESM6_ESM.pdf]

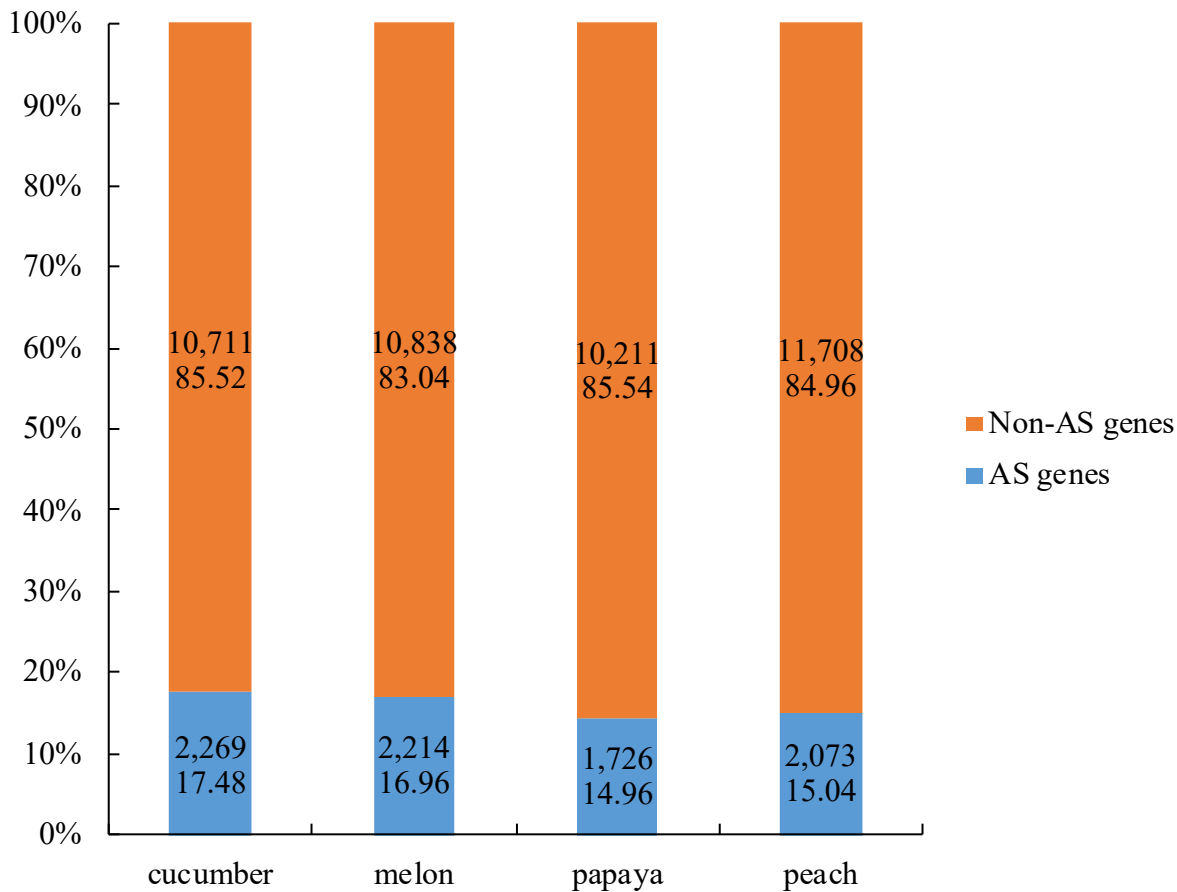

Supplement: Supplementary file 7 — Additional file 7: Fig. S5. Distribution of multiexon genes under alternative splicing. [file 12864_2021_8111_MOESM7_ESM.pdf]

A

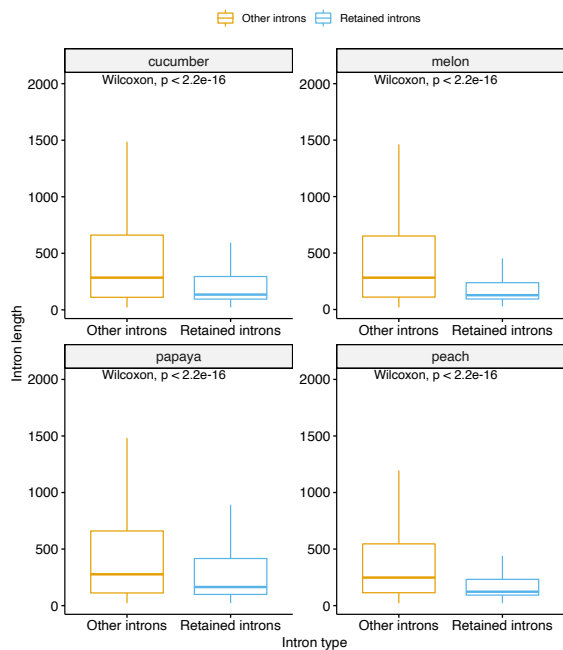

B

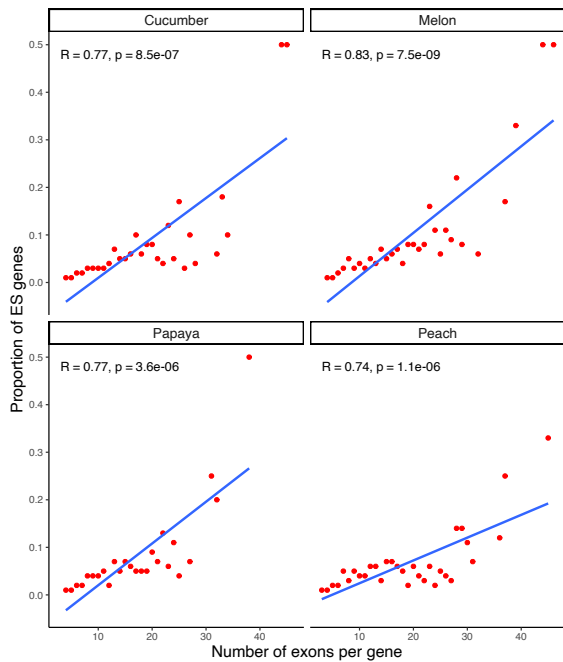

C

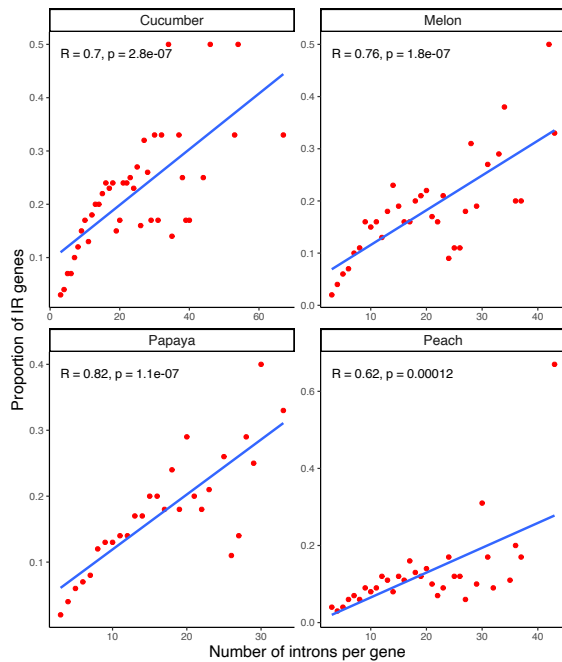

Supplement: Supplementary file 8 — Additional file 8: Fig. S6 Relationship between the genic features and the alternative splicing (AS). a Comparison of the length of introns between retained introns and other introns. b Correlations between the number of exons and the ratio of ES genes. c Correlations between the number of introns and the ratio of IR genes. [file 12864_2021_8111_MOESM8_ESM.pdf]
